# Clinical Named Entity Recognition From Chinese Electronic Health Records via Machine Learning Methods

**DOI:** 10.2196/medinform.9965

**Published:** 2018-12-17

**Authors:** Yu Zhang, Xuwen Wang, Zhen Hou, Jiao Li

**Affiliations:** 1 Institute of Medical Information and Library Chinese Academy of Medical Sciences Peking Union Medical College Beijing China

**Keywords:** clinical named entity recognition, machine learning, treatment, diagnosis, physical examination, human body, syndrome, electronic health records, bidirectional LSTM-CRF

## Abstract

**Background:**

Electronic health records (EHRs) are important data resources for clinical studies and applications. Physicians or clinicians describe patients’ disorders or treatment procedures in EHRs using free text (unstructured) clinical notes. The narrative information plays an important role in patient treatment and clinical research. However, it is challenging to make machines understand the clinical narratives.

**Objective:**

This study aimed to automatically identify Chinese clinical entities from free text in EHRs and make machines semantically understand diagnoses, tests, body parts, symptoms, treatments, and so on.

**Methods:**

The dataset we used for this study is the benchmark dataset with human annotated Chinese EHRs, released by the China Conference on Knowledge Graph and Semantic Computing 2017 clinical named entity recognition challenge task. Overall, 2 machine learning models, the conditional random fields (CRF) method and bidirectional long short-term memory (LSTM)-CRF, were applied to recognize clinical entities from Chinese EHR data. To train the CRF–based model, we selected features such as bag of Chinese characters, part-of-speech tags, character types, and the position of characters. For the bidirectional LSTM-CRF–based model, character embeddings and segmentation information were used as features. In addition, we also employed a dictionary-based approach as the baseline for the purpose of performance evaluation. Precision, recall, and the harmonic average of precision and recall (F1 score) were used to evaluate the performance of the methods.

**Results:**

Experiments on the test set showed that our methods were able to automatically identify types of Chinese clinical entities such as diagnosis, test, symptom, body part, and treatment simultaneously. With regard to overall performance, CRF and bidirectional LSTM-CRF achieved a precision of 0.9203 and 0.9112, recall of 0.8709 and 0.8974, and F1 score of 0.8949 and 0.9043, respectively. The results also indicated that our methods performed well in recognizing each type of clinical entity, in which the “symptom” type achieved the best F1 score of over 0.96. Moreover, as the number of features increased, the F1 score of the CRF model increased from 0.8547 to 0.8949.

**Conclusions:**

In this study, we employed two computational methods to simultaneously identify types of Chinese clinical entities from free text in EHRs. With training, these methods can effectively identify various types of clinical entities (eg, symptom and treatment) with high accuracy. The deep learning model, bidirectional LSTM-CRF, can achieve better performance than the CRF model with little feature engineering. This study contributed to translating human-readable health information into machine-readable information.

## Introduction

### Background

Electronic health records (EHRs) comprise individuals’ health information such as laboratory test results, diagnosis, and medications. This information includes various data types, from structured information such as laboratory test results consisting of test items and the corresponding values, to unstructured data such as clinical narratives in discharge notes [1]. Benefiting from the development of big data techniques, large-scale EHR data mining has become widely used in data-driven medical studies, clinical decision making, and health management. However, plenty of key information on health care is buried in the large amount of unstructured narratives, which makes it difficult to be analyzed computationally. Therefore, clinical named entity recognition (CNER), which is used to identify the boundary of clinical entities such as body parts and diagnoses and then classify them into predefined categories, has been extensively used to extract structured information automatically from English and Chinese EHRs [2-4].

Early named entity recognition (NER) systems often use rule-based approaches that rely on various dictionary resources. More recently, machine learning (ML)–based approaches have been applied to NER, such as maximum entropy (ME), conditional random fields (CRF), support vector machines (SVM), structural support vector machines (SSVM), and multiple deep learning methods [5-10]. Liu et al [11] employed a CRF model based on multiple features including bag-of-characters (BOC), part-of-speech (POS), dictionary, and word-clustering features to identify clinical entities from EHRs. Experiments on 220 clinical tests with different feature combinations showed that a CRF model based on the combination of features including POS features, dictionary features, as well as word clustering features achieved the best performance with an F1 score of 0.8915. Liang et al [12] proposed a novel cascade-type method, which integrated the sentence category classifier from an SVM and the CRF-based clinical entity recognition, to recognize drug names from 324 Chinese admission notes. Their approach achieved an F1 score of 0.935 for the recognition of traditional Chinese medicine drug names and 0.917 for Western medicine drug names. Lei et al [2] systematically investigated the effects of different types of features and different ML models (including CRF, SVM, ME, and SSVM) for CNER on Chinese EHRs. Experiments on their manually annotated corpus of 400 discharge summaries and 400 admission notes showed that both the “word segmentation” feature and the “section information” feature improved the performance of CNER. In addition, among the ML models, SSVM achieved the best performance with an F1 score of 0.9001 and 0.9352 on discharge summaries and admission notes, respectively.

Traditional ML-based approaches such as CRF can achieve good performance on the sequence-labeling tasks but usually rely heavily on hand-engineered features and medical knowledge. However, deep learning methods such as Convolutional Neural Network (CNN) and Recurrent Neural Networks (RNN) can achieve state-of-the-art performance with little feature engineering. Wu et al [8] applied a deep neural network, developed by Ronan Collobert [13], on the CNER task in Chinese clinical text with only word embeddings, achieving an F1 score of 0.9280. Lample et al [14] proposed a bidirectional long short-term memory (LSTM-CRF) model for NER, which achieved an F1 score of 0.9094 on the CoNLL-2003 test set with word embeddings from supervised and unsupervised learning. Misawa et al [15] proposed a “character based” CNN-bidirectional LSTM-CRF model to extract entities from the Japanese Mainichi newspaper corpus. In their model, a CNN model was first used to extract subword information from Japanese characters, and then, the extracted subword information concatenated with the word embedding was fed into a bidirectional LSTM-CRF model to identify entities. Zhu et al [16] developed an end-to-end deep learning model, named GRAM-CNN, for CNER tasks, in which a modified CNN model was first employed to extract local features around a word, and then, a CRF layer was used to model labels jointly based on the output of GRAM-CNN. Their model achieved an F1 score of 87.26% on the Biocreative II dataset. Hu et al [17] built a vote-based hybrid system for the China Conference on Knowledge Graph and Semantic Computing (CCKS) 2017 CNER challenge task, which received the first place with an F1 score of 0.9102. Their hybrid system integrated 4 individual models, including (1) a rule-based model; (2) a CRF model; and (3) 2 bidirectional LSTM models, a conventional bidirectional LSTM model based on word embeddings and a modified bidirectional LSTM model with a fully connected layer added after the LSTM layer to concatenate some hand-crafted features with the LSTM outputs. A total of 4 models were deployed independently for the CNER task with corresponding F1 scores of 0.8682, 0.8969, 0.9017, and 0.8957, respectively. Finally, a vote-based approach was used to combine their results: an entity is selected only when it has been predicted by at least two methods. However, the hybrid system takes considerable time and effort for feature engineering, model constructing, and parameter tuning.

Most of the previous studies on CNER primarily focus on English clinical texts. Various ML models have shown significant performance on CNER on English EHRs. Compared with English CNER, Chinese CNER faces more obstacles and still remains a challenge, which may due to the following reasons: (1) few open access Chinese EHR corpora; (2) a small number of Chinese medical dictionaries and ontology libraries; and (3) complicated properties of the Chinese language, such as the lack of word boundaries, the complex composition forms, and word forms remaining unchanged in all kinds of tense or POS [18,19]. Until the recent 2 years, the number of studies on Chinese CNER has increased rapidly, boosting the performance of the models on Chinese CNER.

### Objectives

In this study, we investigate 2 automatic methods, bidirectional LSTM-CRF and the CRF model, in terms of simultaneously identifying 5 types of clinical entities from Chinese EHR data. Experiment results indicate that the 2 ML models showed significant performance on each type of entity, demonstrating their effectiveness in recognizing multiple types of clinical entities for further data-driven medical research. Our bidirectional LSTM-CRF model can capture not only the past and future input features through the bidirectional LSTM layer but also the sentence-level tag information via the CRF layer. Its performance is comparable with the Top 1 system (F1 score 0.9043 vs 0.9102) in the CCKS 2017 CNER challenge task and better than that of each of the 4 individual models of the Top 1 hybrid system, which needs much effort for feature engineering and model constructing. The bidirectional LSTM-CRF model achieves state-of-the-art performance by utilizing only the character and segmentation information, which significantly alleviates the human work involved in feature engineering to a large extent.

## Methods

### Datasets

A total of 2 datasets were used in this study, the first one is an annotated corpus, which is used for training and testing, whereas the second one, regarded as the development set, is an unlabeled corpus for learning character embedding. All data are derived from the progress notes and examination results of in-patients’ EHRs released by the CCKS 2017 CNER challenge task [20]. The first dataset involves 400 patients’ EHR data, and for each patient, it contains 4 data fields, including (1) general items: usually contain the patient’s demographics and the reasons for admission; (2) medical history: consists of the patients’ past disease history and corresponding treatment, the reasons for current admission, outpatient test results with diagnosis and treatment, and the tests after hospitalization; (3) diagnosis and treatment: mainly include the tests after hospitalization, corresponding diagnosis, and detailed treatment and body condition after treatment (if worse or new symptoms appear, test once again; corresponding diagnosis and treatment will be contained); and (4) discharge note: involves patients’ complaints about their body condition, final tests before discharge, and the doctor’s summary of the patients’ body condition. Moreover, for each field, 5 types of clinical entities—symptom, test, diagnosis, treatment, and body part—were annotated. An example of the original EHR text is shown in Textbox 1 and its manually annotated gold standard provided by the CCKS organizer is shown in Table 1, in which pos_b and pos_e denote the start and end position of the entity in the text. In summary, a total of 10,142 symptoms, 12,689 tests, 1275 diagnoses, 1513 treatments, and 13,740 body parts were annotated in the first dataset. The first dataset was further divided into 2 parts, 300 patients’ data as the training set and 100 patients’ data as the test set. The distribution of entities among the training and test sets is shown in Table 2.

The second dataset includes 2605 patients’ unlabeled EHR data, which was used for learning character embeddings. Character embeddings were trained using individual Chinese characters.

### Dictionary-Based Clinical Named Entity Recognition

Traditionally, dictionary-based CNER approaches utilize medical dictionary resources such as the Unified Medical Language system, Medical Subject Headings, and RxNorm. For Chinese clinical entity recognition, we constructed a new dictionary on the basis of the Chinese Unified Medical Language System (CUMLS) [21] and the training corpus. CUMLS is a knowledge organization system with more than 30,000 medical subject headings and 100,000 medical terms, which incorporate more than 10 thesauruses, taxonomies, glossaries, and medical corpora. In this study, we only choose 54 categories of medical terms, which are related to the 5 types of entities in this study and classify them into 5 predefined categories manually (in terms of the clinical entity types) to build our dictionary. Overall, 6 categories of medical terms from CUMLS are classified as “diagnosis,” 22 categories as “test,” 9 categories as “body part,” 4 categories as “symptom,” and 13 categories as “treatment.” Finally, we construct the dictionary by integrating all the clinical named entities, derived from the annotated labels provided by the CCKS organizer, in the 300 training set with the selected part of the CUMLS. The dictionary we build contains not only the medical terms from medical vocabulary but also the terms from the clinical text, which makes it more suitable for CNER tasks. Maximum forward matching was adopted while extracting clinical entities based on our dictionary.

### Machine Learning Methods for Clinical Named Entity Recognition

CNER is generally converted into a sequence-labeling problem or a classification problem. Sequence-labeling problem means, given a sequence of input tokens A=(a_1_,...,a_n_) and a predefined set of labels L, determine a sequence of labels B=(b_1_,…,b_n_) with the largest joint probability for the sequence of input tokens [22] (b_i_ ∈ L for 1 ≤ i ≤ n). Classification problem means for each input token x, determine the label with the highest probability of classification among the predefined set of labels L. As for CNER, the labels incorporate 2 concepts, the type of the clinical entity and the position of the token within the entity. In this study, we utilize the typical “BIO” labels [23] to represent the position of the tokens within the entities. In BIO labels, B means the token is the beginning of an entity, I means the token is inside an entity, and O means the token is outside of an entity. As there are 5 types of entities, we have overall 11 labels including 5 B classes (B-symptom, B-test, B-diagnosis, B-treatment, and B-body) and their corresponding I classes (I-symptom, I-test, I-diagnosis, I-treatment, and I-body) and O. For instance, a body part entity “心房” is made up of 2 Chinese characters “心” and “房” that are annotated with label B-body and I-body, respectively. In the following sections, we introduce the sequence-labeling algorithm CRF as well as the deep learning models (bidirectional LSTM-CRF) for CNER.

**An example of the original electronic health record text.**

1、患儿为4岁儿童，起病急，病程短。2、以咳嗽，发热为主症。3、查体：咽部稍充血，双扁桃体稍肿大。双肺呼吸音粗，可闻及中小水泡音，结合胸片故诊断为：支气管肺炎。给予静点头孢哌酮、炎琥宁联合抗感染、雾化吸入布地奈德、沙丁胺醇减轻气道高反应 (1. Patient is a 4-year-old children, acute onset, short duration. 2. Main symptoms are cough and fever. 3. Examination: the throat is slightly congestive, double tonsils are slightly swollen. Lung breath sounds thick, a small and medium-sized bubble sound can be heard. Combined with the chest x-ray, diagnosed as: bronchopneumonia. Given cefoperazone combined with andrographolide for anti-infection, aerosolized inhaled budesonide and salbutamol to reduce airway hyperresponsiveness.)

**Table 1:** An example of the manually annotated golden standard.

Entity	pos_b^a^	pos_e^b^	Entity type
咳嗽 (cough)	21	22	Symptom
发热 (fever)	24	25	Symptom
查体 (examination)	32	33	Test
咽部 (throat)	35	36	Body part
充血 (congestion)	38	39	Symptom
双扁桃体 (double tonsils)	41	44	Body part
肿大 (swollen)	46	47	Symptom
双肺 (lung)	49	50	Body part
呼吸音 (breath sound)	51	53	Test
胸片 (chest x-ray)	67	68	Test
支气管肺炎(bronchopneumonia)	74	78	Diagnosis
头孢哌酮 (cefoperazone)	84	87	Treatment
炎琥宁 (andrographolide)	89	91	Treatment
布地奈德 (budesonide)	102	105	Treatment
沙丁胺醇 (salbutamol)	107	110	Treatment
气道 (airway)	113	114	Body part

^a^pos_b: start position.

^b^pos_e: end position.

**Table 2:** Distribution of entities among the training set and the test set.

Dataset	Number of patients	Body part	Diagnosis	Symptom	Test	Treatment	Total
Training set	300	10,719	722	7831	9546	1048	29,866
Test set	100	3021	553	2311	3143	465	9493
All	400	13,740	1275	10,142	12,689	1513	39,359

#### Conditional Random Fields–Based Clinical Named Entity Recognition

CRF is a probabilistic undirected graphical model, which was first proposed by Lafferty in 2001 [24]. It overcomes the shortcomings of the Hidden Markov Model and also solves the label-bias problem of the Maximum Entropy Markov Model. As it takes into account the joint probability distribution of the output sequence of labels, it has been widely used for sequence labeling tasks such as POS tagging, Chinese word segmentation, NER, and CNER. The CRF model decodes the sequence-labeling problem by undirected Markov chain and the Viterbi algorithm with the training criteria: maximize the likelihood estimation of conditional probability of the output sequence of labels Y given the input sequence X. In this study, X is the random variable over the input Chinese characters sequence and Y is the random variable over the corresponding label sequence.

Let P(Y|X) be a linear chain conditional random field. Under the condition that the value of random variable X is x (eg, “患者左腹压痛; patients with left abdominal pressing pain”), the conditional probability of which the random variable Y is y (eg, “O, O, B-body, I-body, B-symptom, and I-symptom”) is defined as:

P(y|x) = 1/Z(x) * exp{ ∑_i,k_ λ_k_ t_k_ (y_i-1_, y_i_, x, i) + ∑_i,l_ μ_l_ s_l_ (y_i_, x, i) }

Z(x) = ∑_y_ exp{ ∑_i,k_ λ_k_ t_k_ (y_i-1_, y_i_, x, i) + ∑_i,l_ μ_l_ s_l_ (y_i_, x, i) },

in which Z(x) denotes the normalization factor, y_i_ (eg, I-symptom) is the label of x_i_ (eg, “痛; pain” in the “患者左腹压痛; patients with left abdominal pressing pain”), and then y_i-1_ (B-symptom) is the label of x_i-1_ “压 (pressing)”. t_k_ (depends on the current label y_i_ and former label y_i-1_) and s_l_ (depends on the current label y_i_) denote the feature functions, and λ_k_ and μ_l_ denote their corresponding weights. Once the corresponding weights are learned, the labels of a new input sequence can be predicted according to P(y|x). The prediction process can be achieved in an efficient way using the Viterbi algorithm. In this study, we use the CRF++ package [25], one of the most popular implementations of CRF, for the implementation of CRF model. The features for training the CRF model are described in the Feature Selection section.

#### Bidirectional Long Short-Term Memory-Conditional Random Fields–Based Clinical Named Entity Recognition

**Figure 1:**
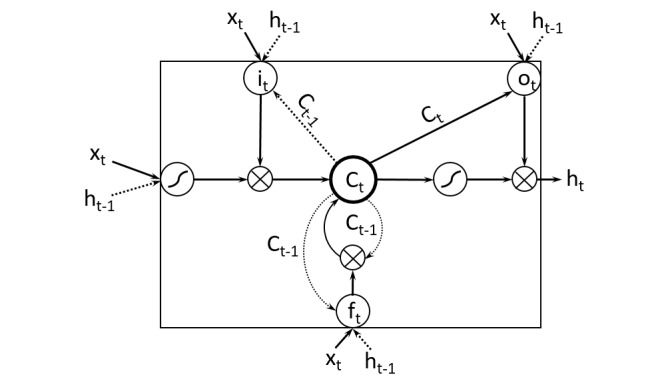
A long short-term memory unit. it: input gate; ft: forget gate; ct: memory cell; ot: output gate; ht: output vector of the LSTM.

Recently, multiple deep neural architectures have been exploited for NER tasks [26-28], among which RNN models usually achieve the best performance, especially the bidirectional LSTM-CRF model. In theory, RNNs are capable of capturing long-distance dependencies; in practice, they fail due to the gradient vanishing or exploding problems. LSTMs are variants of RNNs designed to cope with these gradient vanishing problems by incorporating a memory cell [29]. Figure 1 shows the structure of an LSTM unit at step t [7]. An LSTM unit contains an input gate i_t_, which controls the proportion of input information to the memory cell; a forget gate f_t_, which controls the proportion of the previous information to forget; a memory cell c_t_, which memorizes the long-distance context information; and an output gate o_t_, which controls the proportion of the output information for the next step. The implementation of the LSTM unit is shown as follows:

i_t_ = σ(W_xi_ x_t_ + w_hi_ h_t-1_ + W_ci_ c_t-1_ + b_i_)

f_t_ = σ(W_xf_ x_t_ + W_hf_ h_t-1_ + W_cf_ c_t-1_ + b_f_)

c_t_ = f_t_ • c_t-1_ + i_t_ • tanh(W_xc_ x_t_ + W_hc_ h_t-1_ + b_c_)

o_t_ = σ(W_xo_ x_t_ + W_ho_ h_t-1_ + W_co_ c_t_ + b_o_)

h_t_ = o_t_ • tanh(c_t_),

where σ denotes the element-wise sigmoid function; • denotes the element-wise product; and b_i_, b_f_, b_c_, and b_o_ denote the bias vectors. W denotes the weight matrix, x_t_ denotes the input vector corresponding to the current Chinese character, and h_t_ denotes the output vector of the LSTM, which represents the context information of the current Chinese characters.

**Figure 2:**
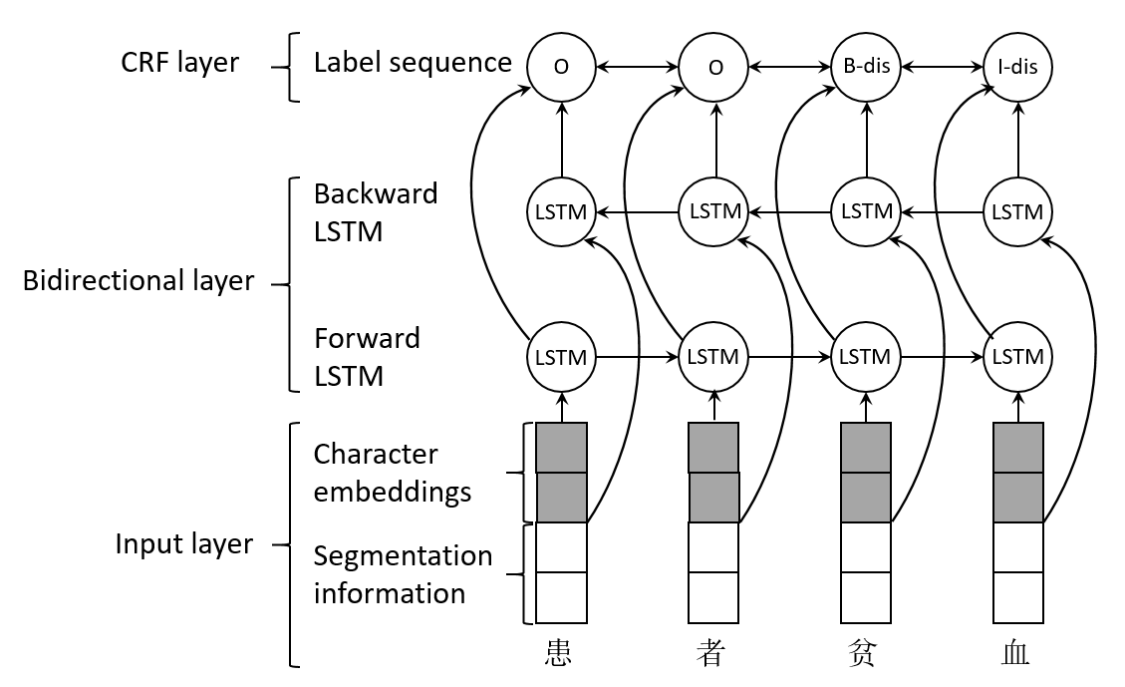
Architecture of the bidirectional long short-term memory-conditional random fields. LSTM: long short-term memory; CRF: conditional random fields; B-dis: B-diagnosis; I-dis: I-diagnosis.

For many sequence-labeling tasks, it is beneﬁcial to have access to both past (left) and future (right) contexts. However, the LSTM’s hidden state h_t_ takes information only from past. Bidirectional LSTM model presents each sequence forward and backward to 2 separate hidden states to capture past and future information, respectively. Then, the 2 hidden states are concatenated to further form the ﬁnal output. Bidirectional LSTM-CRF model, which takes advantage of both bidirectional LSTM and CRF, can simultaneously utilize the past and future input features through the forward and the backward LSTM layer and the sentence level tag information via the CRF layer. The architecture of the bidirectional LSTM-CRF is shown in Figure 2, which consists of an input layer, 2 LSTM layers, and a CRF layer.

When predicting the tags of Chinese characters, first, given a sentence S=(c_1_,...,c_n_), each character c_t_ (1≤t≤n) is represented by vector x_t_ (the concatenation of the character embeddings and the segmentation information) generated in the input layer. Second, the forward and the backward LSTM layer take the sequence of character representations X=(x_1_,...,x_n_) as input and generate the representation of the left (h_l_=h_l1_,...,h_ln_) and right (h_r_=h_r1_,...,h_rn_) context for each character, respectively. Third, the sequence of overall context representations is h=(h_1_,...,h_n_), where h_t_ is the concatenation of h_lt_ and h_rt_. Finally, the sequence of overall context representations is taken as input for the CRF layer to predict the output label sequence L=(l_1_,...,l_n_).

### Feature Selection

For training the CRF model, we select 4 types of features, BOC, POS tags, character types (CT), as well as the position of the character in the sentence (POCIS). NLPIR Chinese word segmentation system Institute of Computing Technology, Chinese Lexical Analysis System (ICTCLAS)-2016 [30] is utilized for word segmentation. While using ICTCLAS-2016 for segmentation, POS tags are generated simultaneously. As we use character-level information instead of word-level information, the POS tag of the Chinese character is just the POS tag of the corresponding word, which contains that character. In addition, we manually classify all the characters in the EHR dataset into 5 CT (including W: common character; D: numbers; L: letters; S: ending punctuation; and P: symmetrical punctuation). To validate the effectiveness of the bidirectional LSTM-CRF model in identifying clinical entities with much less feature engineering than the CRF model, different combinations of features are fed into the CRF model.

For training the bidirectional LSTM-CRF model, we employ the character embeddings and segmentation information as our features. Character embeddings are learned through Google’s word2vec [31] on the 2605 patients’ unlabeled dataset. The segmentation information is generated by the Jieba segmentation system [32].

### Experiment

#### Experimental Setup

We conducted an experimental study to compare ML-based CNER with the dictionary-based approach. First, we divide the first dataset into 2 parts, the first part, which contains 300 patients’ EHR data, for training, and the second part, which involves 100 patients’ EHR data, for testing.

In the CRF model, the content window size is set to 5 for extracting character features, including the 2 preceding characters, the current character, and the 2 following characters. Different combinations of features have been tried to train the CRF model, including (1) BOC; (2) BOC+POS tags; (3) BOC+POS tags+CT; and (4) BOC+POS tags+CT+POCIS. In addition, we applied 10-fold cross validation for tuning model parameters. In 10-fold cross validation, the training set was randomly divided into 10 parts; each time, we used 1 part as the test set and the remaining 9 parts as the training set for the experiment. Finally, we used the average F1 score of the 10 experiments to estimate the accuracy of the model and tune the parameters.

As for the deep learning model, we fix the learning rate at 0.0004, the dropout at 0.5, and the character embedding dimension at 100. The number of hidden units in bidirectional LSTM-CRF is set to 100, and the optimizer is set to Adam.

As for dictionary-based CNER, which is regarded as the baseline approach, maximum forward matching based on our dictionary is adopted for extracting clinical entities.

#### Evaluation Criteria

The evaluation for this CNER challenge task is implemented through the algorithm provided by CCKS 2017 organizers, which reports the Precision (P), Recall (R), and F1 score for all clinical entities using exact matching methods [33]. According to the algorithm, we define O=(O_1_,...,O_m_) as the output set of the system and G=(G_1_,...,G_n_) as the manually annotated set (in terms of the golden standard) provided by the task organizer. Then o_*i*_ ϵ *0* and g_i_ ϵ *G* are strictly equivalent only when:

o_i_.mention = g_j_.mention

o_i_.pos_b = g_j_.pos_b

o_i_.pos_e = g_j_.pos_e

o_i_.category = g_j_.category

Here, mention represents the content of the entity, pos_b and pos_e separately denote the start and end position of the entity in the EHR text, and category represents the entity type. On the basis of the above equivalence relation, strict evaluation metrics are implemented as follows:

P=|S ∩ G| / |S|

R=|S ∩ G| / |G|

F1 = 2PR / (P+R)

## Results

To validate the effectiveness of the ML models on simultaneously identifying various types of clinical entities from Chinese EHRs, we carried out comparative experiments on the basis of CCKS CNER corpus.

As shown in Table 3, the best overall performance was achieved by the bidirectional LSTM-CRF model with an F1 score of 0.9043, followed by the CRF models with F1 scores from 0.8547 to 0.8949, and finally the dictionary-based model with an F1 score of 0.5924. ML models achieved significantly better performance than the dictionary-based approach. In addition, with the number of features increasing, performance of the CRF model continued to improve, increasing F1 score from 0.8547 to 0.8949. However, even the best CRF model with all 4 types of features was slightly worse than the bidirectional LSTM-CRF model.

**Figure 3:**
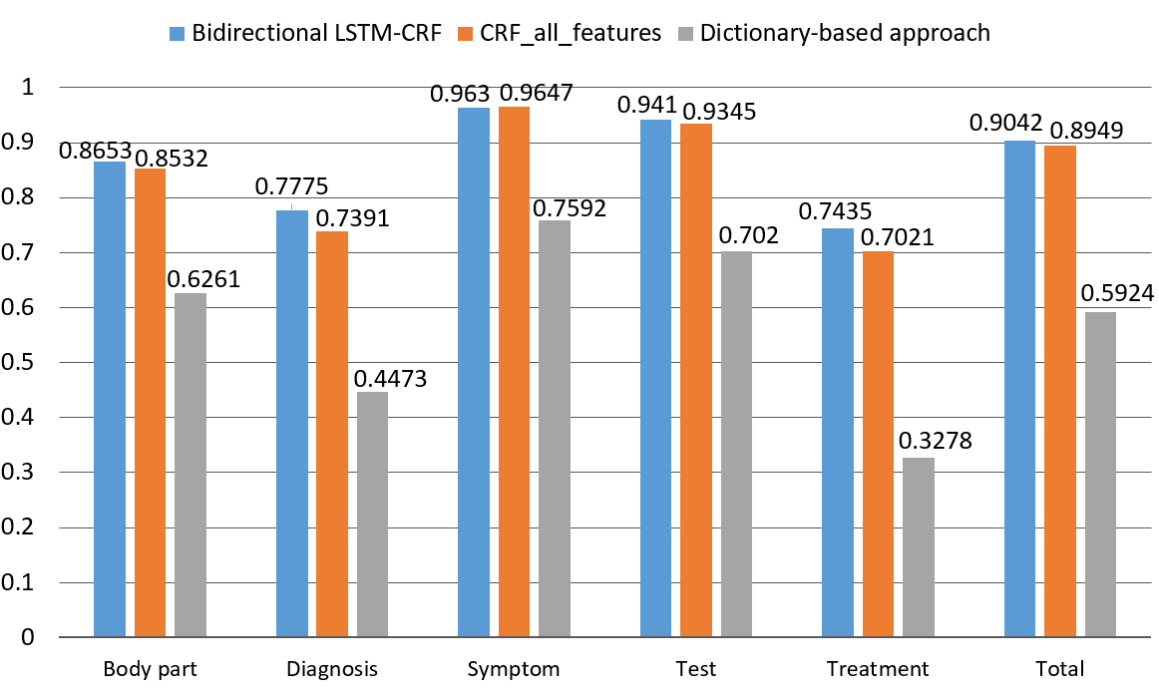
Comparison of F1 scores between dictionary-based approach and machine learning–based approaches among 5 entity types; LSTM-CRF: long short-term memory-conditional random fields; CRF: conditional random fields.

Besides the overall performance, Table 4 showed the detailed performance of the ML models as well as the dictionary-based model on each type of clinical entity. The bidirectional LSTM-CRF model achieved the highest recalls in all the 5 types of clinical entities, whereas the CRF model always achieved the highest precisions in each type of entity except for “treatment” type. Among the 5 types of entities, the “symptom” type of entities had the best performance with an F1 score over 0.96 in ML models, followed by the “test” type of entities with an F1 score around 0.94, whereas the “treatment” type of entities always received the worst performance with an F1 score less than 0.75. Furthermore, Figure 3 more intuitively shows the comparison of the detailed performance between the ML-based models and the dictionary-based approach.

**Table 3:** Overall performance of the bidirectional long short-term memory-conditional random fields model, conditional random fields–based models with different feature combinations, and the dictionary-based model.

Model	Precision	Recall	F1 score
Dictionary-based model	0.5215	0.6855	0.5924
CRF^a^ model+BOC^b^	0.8792	0.8316	0.8547
CRF model+BOC+POS^c^ tags	0.9065	0.8529	0.8789
CRF model+BOC+POS tags+CT^d^	0.9144	0.8658	0.8895
CRF model+BOC+POS tags+CT+POCIS^e^	0.9203	0.8709	0.8949
Bidirectional LSTM-CRF^f^ model	0.9112	0.8974	0.9043

^a^CRF: conditional random fields.

^b^BOC: bag-of-characters.

^c^POS: part-of-speech.

^d^CT: character types.

^e^POCIS: position of the character in the sentence.

^f^LSTM-CRF: long short-term memory-conditional random fields.

**Table 4:** Detailed performance of the bidirectional long short-term memory-conditional random fields–based, conditional random fields–based, and dictionary-based clinical named entity recognition approaches.

Entity type	Bidirectional LSTM-CRF^a^			CRF^b^_all_features			Dictionary-based approach		
	Precision	Recall	F1 score	Precision	Recall	F1 score	Precision	Recall	F1 score
Body part	0.8873	0.8444	0.8653	0.8909	0.8186	0.8532	0.6081	0.6452	0.6261
Diagnosis	0.8086	0.7486	0.7775	0.8148	0.6763	0.7391	0.3545	0.6058	0.4473
Symptom	0.9584	0.9675	0.9630	0.9715	0.9580	0.9647	0.7591	0.7594	0.7592
Test	0.9314	0.9510	0.9411	0.9459	0.9233	0.9345	0.7093	0.6949	0.7020
Treatment	0.7833	0.7075	0.7435	0.7581	0.6538	0.7021	0.2240	0.6108	0.3278
Total	0.9112	0.8974	0.9043	0.9203	0.8709	0.8949	0.5215	0.6855	0.5924

^a^LSTM-CRF: long short-term memory-conditional random fields.

^b^CRF: conditional random fields.

## Discussion

### Principal Findings

Essentially, recognizing various types of clinical entities allows extraction of the structured information of patients, which can be further exploited for data-driven medical research, clinical decision making, and health management. Compared with previous studies in CNER, ML-based methods can simultaneously extract 5 types of entities. Moreover, the proposed bidirectional LSTM-CRF model achieves a performance that is comparable with the Top 1 system, which is an ensemble model incorporating 4 ML models including a rule-based model, a CRF model, and 2 RNN models, in the CNER challenge only using character embeddings, and the segmentation information, therefore, reduces considerable efforts for feature engineering and model constructing.

### Dictionary-Based Clinical Named Entity Recognition Versus Machine Learning–Based Clinical Named Entity Recognition

Experiments on the CCKS 2017 CNER challenge corpus show that ML-based models (bidirectional LSTM-CRF and CRF) achieve remarkably better performance than the dictionary-based method. Different from the maximum forward matching of the dictionary-based CNER, ML methods can sufficiently exploit the context information (eg, bag of Chinese characters and context representation information derived from LSTM), syntactic information (eg, POS tags), and structure information (eg, the position of the Chinese character in the sentence), which makes their performance significantly better. Furthermore, the performance of ML models is comparable with the Top 1 system in the CNER challenge with an overall F1 score of 0.9102, validating the effectiveness of the 2 ML-based methods in simultaneously recognizing multiple types of clinical entities for further data-driven medical studies.

### Bidirectional Long Short-Term Memory-Conditional Random Fields Versus Conditional Random Fields

The bidirectional LSTM-CRF model achieves the best overall performance (see Table 3) but only utilizes the character embeddings and the segmentation information. Compared with the traditional CRF model, bidirectional LSTM-CRF not only takes advantage of CRF but also receives the benefits of bidirectional LSTM, which can generate long-distance context representations from the past and future input features. For example, given an input sequence of Chinese characters “生化检查: 谷丙转氨酶23.4 U/L, 谷草转氨酶21.7 U/L...葡萄糖5.78 mmol/L (biochemical tests: alanine aminotransferase 23.4 U/L, aspartate aminotransferase 21.7 U/L...glucose 5.78 mmol/L),” when predicting the labels of “葡萄糖 (glucose),” LSTM can capture the long-distance context information “生化检查 (biochemical tests)” and take it into labels prediction, which may make the prediction of the labels of “葡萄糖 (glucose)” be “B-test, I-test, I-test” rather than “B-treatment, I-treatment, I-treatment.”

Furthermore, by comparing the results of CRF models and the bidirectional LSTM-CRF model in Table 3, we find that, given the same features, bidirectional LSTM-CRF model performs obviously better than the CRF model. Even with more features, the CRF model is still slightly worse than the bidirectional LSTM-CRF model. The bidirectional LSTM-CRF model has a remarkable advantage in taking little effort for feature engineering to get higher efficiency and more robust performance in different types of entity recognition. However, the CRF model can also perform well in CNER but requires elaborate feature engineering and, thus, lacks efficiency, scalability, and generality. In brief, similar to NER in other domains, deep learning models such as bidirectional LSTM-CRF show great potential on CNER in the medical domain, outperforming the traditional state-of-the-art method CRF, which involves massive feature engineering.

### Differences Among the Performance of Five Types of Entities

Despite the impressive overall performance, the ML models do not show superiority over all the 5 types of clinical entities. As shown in Figure 3, among the 5 types of entities, the “symptom” type of entities achieve the best performance, followed by the “test” and “body part,” whereas the performance of “diagnosis” and “treatment” is approximately 10% lower than that of the other 3 types. This may be due to 2 reasons: (1) the number of “diagnosis” type entities and “treatment” type entities is almost 10 times less than the other type of entities, as shown in Table 1, and fewer training samples limited the recognition effect and (2) a clinical entity may be annotated as different entity types in different contexts. For example, “头痛” (headache) is annotated as the “symptom” type in the context of “发作性头痛、头晕6年” (paroxysmal headache and dizziness for 6 years) but annotated to the “diagnosis” type in the context of “间断性头痛2周” (intermittent headache for 2 weeks). Incorporating medical domain information into the ML-based models and making a larger training set may help solve the problem.

### Error Analysis

An error analysis on our 2 ML-based models shows that plenty of errors often occur when predicting tags on long entities with composite structures. For example, “高血压病腔隙性脑梗死” (hypertension Lacunar Cerebral Infarction), which is annotated as a “diagnosis” type entity in the golden standard, is automatically annotated as 2 entities “高血压” (hypertension) and “腔隙性脑梗死” (lacunar infarction) in our ML models. Especially, we find that, in the EHR text, a “body part” type of entity is often followed by a “symptom” or a “diagnosis” type of entity, which makes it difficult to identify the border between the 2 entities. For instance, in EHR text “股骨骨折 (femoral fracture),” the “body part” type of entity “股骨 (femur)” is followed by a “symptom” type of entity “骨折 (fracture).” Incorporating domain knowledge and medical dictionaries as well as combining the active learning methods with current ML models and increasing the scale of datasets might be the right path.

Furthermore, taking CRF model based on all features (BOC, POS tags, CT, and POCIS) as an example, we conduct an in-depth error analysis on its result to explore the effectiveness and limitations of the ML models on Chinese CNER either from a statistical view or from the clinical view. Table 5 shows the distribution of different types of errors as well as some examples, in which “GT-P” denotes the entities that were not identified by CRF; “P-GT” represents the entities recognized by CRF but are not in the ground truth; and “INTERSECT” denotes that for each entity, there is intersection between the ground truth and the entity predicted by CRF, for example, when extracting entities on EHR text “患者有脂肪肝病史 (the patient has a history of fatty liver),” the entity recognized by CRF is “肝 (liver),” having intersecting part “肝 (liver)” with the ground truth “脂肪肝 (fatty liver).” Overall, there are 1386 errors, 143 (10.32%) errors with type “GT-P,” 604 (43.58%) errors with type “P-GT,” and 639 (46.10%) errors with type “INTERSECT.”

As for “GT-P” type of errors, only 1.51% (143/9493) entities of the test set are missed by the CRF model, which demonstrate its effectiveness in Chinese CNER. After further analysis on type “GT-P” errors from a medical view, we find that some entities missed by CRF model, which may be because the ground truth is not accurate, contain some punctuations that are not related to the entities. For example, the ground truth “肿，(swollen,)” should be “肿 (swollen)” rather than “肿 (swollen)” with punctuation “,”. Moreover, some entities such as “对称 (symmetry)” do not belong to each type of clinical entity from the clinical view and should not appear in the ground truth. These entities are not recognized by the CRF model, which is not a problem of the model but a problem of ground truth. With more accurate ground truth, our results can be better. Moreover, some errors such as the “Symptoms and signs” type of new entities “活动障碍” (activity disorder), “听力下降” (hearing loss), and “功能障碍” (dysfunction) were not recognized by CRF, which may be because they never appear in the training set. Without sufficient training examples, it is challenging to effectively identify clinical entities, especially the unknown ones, for supervised ML models. Some studies [34-36] have attempted to apply unsupervised ML methods to recognize entities from clinical text on the basis of lexical resources, syntactic knowledge, and corpus statistics. It is worth making further efforts in Chinese clinical entity recognition using the unsupervised methods when lacking training data.

In addition, through the analysis on “P-GT” type of errors, we find that most of the entities in these types of errors are clinically meaningful, such as “2型糖尿病 (type 2 diabetes)” and “冠心病 (coronary disease).” These entities recognized by the CRF model should be the ground truth rather than errors. The reason behind this may be due to missing annotations while manually building the ground truth. Thus, these type of “errors” should be the advantage of our models, which could maintain high efficiency and accuracy during CNER, rather than errors. Moreover, some entities such as “腔隙性脑梗 (lacunar clog)” are new entities that never appeared in the ground truth. These entities are meaningful to clinicians and should be recognized. This proves that our model has the ability to identify a few new clinical entities from Chinese EHR. However, some entities recognized by our models, such as “比重 (proportion),” do not make any sense.

Finally, the deep analysis of “INTERSECT” type of errors shows that most of the errors are due to the different granularities between our results and the ground truth. For example, the ground truth for clinical text “患者于去年诊断为脑水肿 (the patient was diagnosed with cerebral edema last year)” is “水肿 (edema)” and our result is “脑水肿 (cerebral edema).” This is a limitation of ML models that cannot accurately identify entities at the appropriate granularity. However, plenty of entities appear to be annotated at different granularities in different EHR documents when building the ground truth. For example, text “脑梗死 (cerebral infarction)” is sometimes annotated as “脑 (brain)” and sometimes annotated as “脑梗死 (cerebral infarction)” and text “右侧丘脑腔隙性脑梗死 (right thalamic lacunar infarction)” is sometimes annotated as “右侧丘脑 (right thalamic)” and “腔隙性脑梗死 (right lacunar infarction),” whereas it is sometimes annotated as “脑梗死 (cerebral infarction).” The ambiguity of the granularities in the ground truth will make the ML models more difficult to extract clinical entities on appropriate granularities. Specific annotation rules on annotation granularities as well as high-quality datasets could be constructed to further improve the performance of ML models on Chinese CNER.

### Future Directions

In the future, we will not only develop new ML methods to enhance the accuracy of CNER but will also try to collect and standardize the recognized entities into the standard medical lexicons. Considering that different types of entities have different distributions in different fields of EHR, for instance, “treatment” type of entities often concentrates on the “diagnosis and treatment” field and rarely appears in the “general items” field, separately building ML-based models on each type of field data rather than on all EHR data may be a worthwhile study. As the amount of Chinese EHR data is limited, incorporating the active learning methods with ML models may be a possible future direction. Furthermore, when such structural patient information is used for data-driven medical studies, the time order of the clinical entities as well as their modifications are usually required. Therefore, a future direction is to identify more details of the clinical entities.

**Table 5:** Distribution of different types of errors in the results of the conditional random fields model based on all the 4 types of features (N=1386).

GT-P^a^ (N=143)	P-GT^b^ (N=604)	INTERSECT^c^ (GT vs P; N=639)
尿蛋白- （urinary protein-）	2型糖尿病 （type 2 diabetes）	右侧丘脑腔隙性脑梗死 versus 右侧丘脑 + 腔隙性脑梗死 （right thalamic lacunar infarction vs right thalamic+lacunar infarction）
低血糖 （hypoglycemia）	冠心病（coronary disease）	胃肠 versus 急性胃肠炎 （stomach and intestine vs acute gastroenteritis）
对称 （symmetry）	胸 （chest）	糖尿病肾病 versus 糖尿病 + 肾病 （diabetic nephropathy vs diabetes+nephropathy）
瞳孔 （pupil）	腔隙性脑梗 （lacunar clog）	右下后 versus 右下后牙 （right lower back vs lower right posterior teeth）
冠心病 （coronary disease）	脂肪肝 （fatty liver）	水肿 versus 脑水肿 （edema vs brain edema）
肿， （swollen,）	比重 （proportion）	氨溴索注射液祛痰 versus 氨溴索注射液 （ambroxol injection to remove phlegm vs ambroxol injection）
胃粘膜 （gastric mucosa）	角膜 （cornea）	皮肤、粘膜 versus 皮肤 + 黏膜 （skin、mucous membrane vs skin+mucous membrane）
寒战 （chill）	脑萎缩 （encephalatrophy）	脂肪肝 versus 肝 （fatty liver vs liver）
无力 （faintness）	峰值 （peak value）	尼群地平药物 versus 尼群地平 （nitrendipine drug vs nitrendipine）
皮肤 （skin）	活动障碍 （activity disorder）	脑 versus 脑梗死 （brain vs cerebral infarction）

^a^GT-P: Entities that were not identified by CRF.

^b^P-GT: Entities recognized by CRF but are not in the ground truth.

^c^INTERSECT: For each entity, there is an intersection between the ground truth and the entity predicted by CRF.

### Conclusions

CNER is one of the basic works of data-driven medical research. However, previous studies usually focused on recognizing a single type of clinical entity. In this study, we implemented 2 ML methods, including the bidirectional LSTM-CRF and the CRF models, for simultaneously recognizing 5 types of clinical entities from the Chinese EHR corpus provided by the CNER challenge of CCKS 2017. Compared with the baseline dictionary-based approach, ML methods show remarkably better performance than the former. Moreover, the deep learning model bidirectional LSTM-CRF, outperforming the traditional CRF model in the overall result, achieves state-of-the-art performance on the basis of the character and segmentation information, which alleviates the human work involved in feature engineering to a large extent.

## Abbreviations

BOC: bag-of-characters
CCKS: China Conference on Knowledge Graph and Semantic Computing
CNER: clinical named entity recognition
CNN: Convolutional Neural Network
CRF: conditional random fields
CT: character types
CUMLS: Chinese Unified Medical Language System
EHRs: electronic health records
ICTCLAS: Institute of Computing Technology, Chinese Lexical Analysis System
LSTM: long short-term memory
ME: maximum entropy
ML: machine learning
NER: named entity recognition
POCIS: position of the character in the sentence
POS: part-of-speech
RNN: Recurrent Neural Networks
SSVM: structural support vector machines
SVM: support vector machines
